# Supplementary material for: Research on Fast and Precise Positioning Strategy of an Ultrasonic Motor Based on the Ultrasonic Friction Reduction Theory
Source: Micromachines (Basel). 2022 Sep 17;13(9):1542. doi: 10.3390/mi13091542 (PMC9505952; doi:10.3390/mi13091542)
Supplement: Supplementary file 1 [file micromachines-13-01542-s001.zip › micromachines-1897209-supplementary.pdf]

Supplementary Material

A The theory of ultrasonic friction reduction in the vertical direction

A.1 Vibration model of stator and friction material in vertical direction

According to the knowledge of tribology, when two objects in contact with each other have a relative movement or a trend of relative movement, there will be friction on the contact surface, resulting in friction and wear. In the experimental study of piezoelectric ultrasonic motor, it is found that if you directly touch the working convex tip with your hand, you will feel the feeling of friction reduction at the contact position of your hand. When the moving frequency of the stator is not high, the stator and the rotor will move up and down together, and at this time, they are in close contact. If the rotor has a certain rotating speed, the surface of the stator and friction material will generate friction; With the increase of the vibration frequency of the stator, although downward force acts on the friction material, the rotor can't move up and down at the same time due to its mass inertia. At this time, the rotor will be "vacated", and the gap distance between the stator and the rotor is δ. If one vibration period of the stator is taken as the research object, the contact state between the stator and the rotor is shown in Figure S1.

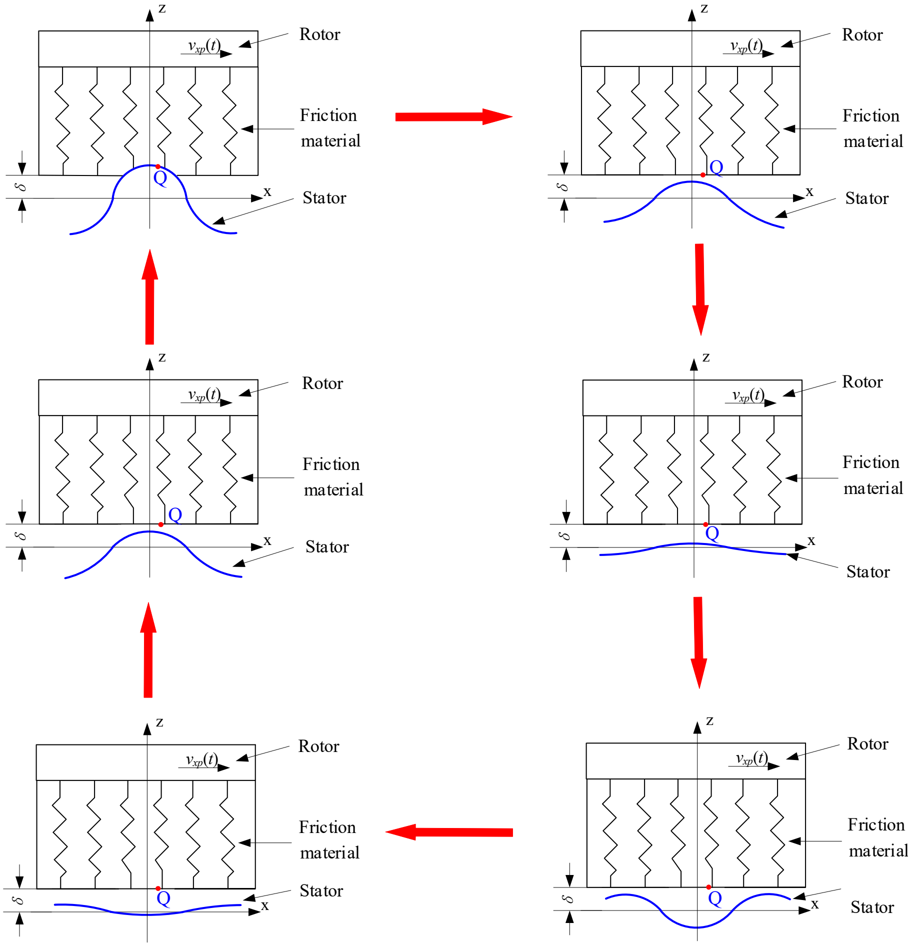

Figure S1. Schematic diagram of ultrasonic friction reduction theory in the vertical direction

A.2 Analysis of vertical ultrasonic antifriction theory

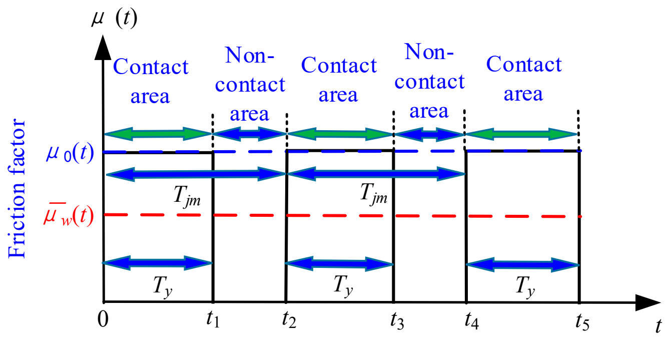

Figure S2. Variation diagram of friction coefficient

From the variation curve in Figure S2, it can be seen that when the stator vibrates at a high frequency in B09 mode, the average friction coefficient of the equivalent friction coefficient when the stator and rotor are in contact with each other decreases (shown by the dashed line in the Figure S2). The average value of the coefficient of friction $\overline{\mu_w}(t)$ for the particle Q of the friction material is given by the following expression.

$$\overline{\mu_w} = \frac{T_y \mu_0}{T_{jm}} \tag{S1}$$

where μ_0 represents the sliding friction coefficient when the stator and the friction material are in complete contact, T_y represents the contact time of the stator and the friction material, and T_{jm} represents one vibration cycle of the stator and the friction material. According to the working principle of the ultrasonic motor, the vibration state of the stator is related to the driving frequency. When the driving frequency is higher, the gap δ distance between the stator and rotor is larger, the contact time between the stator and the friction material is shorter, the average value of the friction coefficient is smaller, and the phenomenon of ultrasonic friction reduction is more obvious.

B Experiment of resistance moment measurement based on ultrasonic friction reduction theory

The positioning method proposed in this paper is based on the principle of ultrasonic friction reduction to drive the motor. In the process of theoretical modeling, the resistance torque of single-phase energization at different driving frequencies needs to be measured, and according to the analysis above, the farther the driving frequency is from the resonant frequency, the larger the equivalent friction coefficient and the larger the resistance torque, and vice versa. To verify the correctness of this theory, the following resistance moment test experiment is conducted as shown in Figure S3.

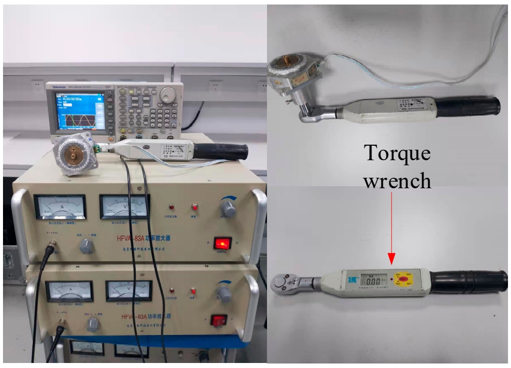

Figure S3. Experimental diagram of the measurement of resistance moment

As shown in Figure S3, firstly, the sinusoidal signals with different frequencies emitted by the signal generator are amplified by the power amplifier to a voltage of 500$V_{P\text{-}P}$, and then applied to the A-phase piezoelectric ceramic sheet of the stator. The pre-pressure of the motor is set to F_c=180N, and the driving frequency setting interval is $2\pi\times41$kHz~$2\pi\times45$kHz; Then fix the motor and adjust the torque wrench to the peak mode to collect data; Finally, the torque wrench is used to rotate the motor shaft, and the measured value of resistance moment with single-phase circular frequency is shown in Figure S4.

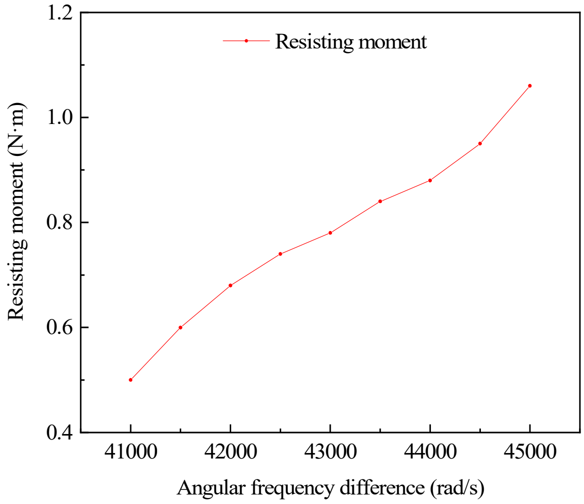

Figure S4. Measured resistance torque as single-phase drive circle frequency varies
